# Editorial: Novel strategies to target cell death signaling in cancer and neurodegenerative diseases: new findings and mechanistic studies

**DOI:** 10.3389/fcell.2024.1383301

**Published:** 2024-02-26

**Authors:** Qi Zhang, Kun Xiong

**Affiliations:** ^1^ Department of Human Anatomy and Neurobiology, School of Basic Medical Science, Central South University, Changsha, China; ^2^ Key Laboratory of Emergency and Trauma of Ministry of Education, Hainan Medical University, Haikou, China; ^3^ Hunan Key Laboratory of Ophthalmology, Changsha, China

**Keywords:** regulated cell death, pyroptosis, apoptosis, necroptosis, PANoptosis, neurodegeneration, cancer

Regulated cell death (RCD) is an evolutionarily conserved process that is essential for organ development and tissue homeostasis maintenance (Hu et al., 2021; Kim et al., 2022; Ploumi et al., 2023). Several key types of RCD have been identified based on distinct cell death signaling pathways, such as apoptosis, necroptosis, pyroptosis, and PANoptosis, which widely participate in the progression and development of human diseases (Zhang et al., 2021; Yan et al., 2022; Zhou et al., 2023; Wan et al., 2024). Cancer and neurodegenerative diseases are two extensively studied human diseases in RCD-related mechanisms and provide new thoughts to other diseases (Yang M. et al., 2022; Yan et al., 2023; Zhang et al., 2023; Ban et al., 2024). Targeting RCD is a viable approach for developing therapeutic strategies, including pharmaceutical discovery and stem cell-based therapy (Yao et al., 2021; Yang Y.-D. et al., 2022; Chen et al., 2023; Hu et al., 2024). The Research Topic, titled “Novel strategies to target cell death signaling in cancer and neurodegenerative diseases: new findings and mechanistic studies” in Frontiers in Cell and Developmental Biology, comprises a series of six articles revealing the molecular mechanisms of different types of RCD in human disease and providing potential candidates for future therapeutic discovery.

Deubiquitinases are a large family of ubiquitin proteases implicated in regulating cell death and various cellular functions (Snyder and Silva, 2021; Zheng et al., 2023). The article by Corno et al. discovered increased USP8 activity in cisplatin-resistant cells through biochemical analysis of paired sensitive and resistant cells. Using colony forming assay, they found that silencing USP8 increased sensitivity to cisplatin in cisplatin-resistant variants and decreased activation of receptor tyrosine kinases. Notably, the authors demonstrated that increased cisplatin sensitivity was associated with an increased rate of apoptosis and enhanced caspase 3/7 activation. Furthermore, they found that 40% of tumors were USP8 positive using immunohistochemical staining based on clinical specimens from patients with advanced-stage ovarian carcinoma, indicating that USP8 could be an independent prognostic factor for adverse outcomes.

Peptidyl-prolyl cis-trans isomerase NIMA-interacting 1 (Pin1) belongs to the family of Peptidyl-prolyl isomerases (PPIases), which convert the peptide bond between amino acids and prolines from cis to trans conformation and *vice versa* (Hu et al., 2020; Biswas et al., 2022). Using bibliometric analysis, Zhang et al. analyzed a total of 395 papers published from 2000 to 2021 and showed an in-depth understanding of the role of Pin1 in the signaling pathways of different types of RCD. Additionally, they indicated that future research could pay more attention to the treatment of cell death-related diseases and disorders with Pin1 inhibitors.

Glaucoma is one of the leading causes of blindness characterized by progressive optic nerve neuropathies and loss of retinal ganglion cells (RGCs) (Ju et al., 2023; Zhao et al., 2023). In their review, Li et al. provided a comprehensive summary of the molecular mechanisms of autophagy in glaucoma and the potential targets of interventions related to autophagy. They also suggested that targeting autophagy and blocking the apoptosis of RGCs could be useful for the treatment of glaucoma in the future.

Retinitis pigmentosa (RP) is a group of inherited retinal dystrophies characterized by the progressive loss of rod photoreceptors and the subsequent degeneration of cone photoreceptors (Bocquet et al., 2023; Nguyen et al., 2023). The article by Huang et al. provided a study of genetic genealogy to identify causative variants in Han-Chinese people with autosomal recessive RP. Using whole exome sequencing and Sanger sequencing, they identified two heterozygous variants in the USH2A gene, c.3304C>T (p.Q1102*) and c.4745T>C (p.L1582P), in the proband, which were inherited from parents and transmitted to the daughters in a six-member, three-generation family with autosomal recessive RP. The findings expand the knowledge of the USH2A gene variants and may contribute to genetic diagnosis and disease management in the field of RP.

In the cerebral cortex, amyloid β (Aβ) and other amyloid proteins can enter cells and their depositions can act together to cause mitochondrial dysfunction and subsequent cell death in different tissues (Sandberg et al., 2022). And, emerging studies found that cerebral amyloid angiopathy (CAA) is often accompanied by cardiac injury, involving amyloid proteins, which can cause the second hit to the heart (Stakos et al., 2020). The review by Xu et al. summarized the recent progress in the field of cerebral amyloid angiopathy-related cardiac injury and highlighted the common pathogenesis and associated impactors in CAA and cardiac amyloidosis and the possible mechanism of CAA affecting heart injury. Regarding the clinical concerns, the authors suggest that the onset age of CAA diagnosis needs to be younger in the future.

SIRT3 is a deacetylase that regulates cellular energy metabolism in mitochondria and is involved in acute brain injuries (Fu et al., 2022). The review by Yang et al. summarized the molecules that regulate SIRT3 to uncover brain-protective mechanisms, conduct further research, and aid drug development. In addition, they highlighted the therapeutic potential of SIRT3 as a target for treating catastrophic brain injury.

In summary, this Research Topic brings new findings and in-depth thinking on cellular and molecular mechanisms involved in the cell death signaling pathways. Moreover, the targets and strategies provided by the papers indicate potential strategies for treating cancer and neurodegenerative diseases affected by cell death.
